# A Comprehensive Review on the Pharmacological Activities and Biosynthetic Strategies of Protocatechuic Acid

**DOI:** 10.3390/life16071206

**Published:** 2026-07-21

**Authors:** Chongde Lai, Hailin Xia, Yuhuan Zhang, Yutong He, Xiaoyu Wu, Bangce Ye, Hui Yang, Bin Zhang

**Affiliations:** 1 Jiangxi Engineering Laboratory for the Development and Utilization of Agricultural Microbial Resources, College of Bioscience and Bioengineering, Jiangxi Agricultural University, Nanchang 330045, China; chongdelai_jxau@163.com (C.L.); xiahailin@jxau.edu.cn (H.X.); 13970482125@163.com (Y.Z.); heyutong@hnu.edu.cn (Y.H.); xywu166@163.com (X.W.); 2 State Key Laboratory of Bioreactor Engineering, East China University of Science and Technology, Shanghai 200237, China; bcye@ecust.edu.cn

**Keywords:** protocatechuic acid, pharmacological activities, microbial cell factories, microbial fermentation

## Abstract

Protocatechuic acid (PCA) is a simple natural phenolic acid widely distributed in plants. It is recognized as an active constituent of numerous traditional herbal medicines and serves as an important metabolic intermediate of polyphenolic compounds such as anthocyanins and proanthocyanidins. At present, PCA production relies on plant extraction and microbial fermentation. Among these, microbial fermentation has emerged as an attractive approach for industrial development owing to its process controllability, environmentally benign nature, and potential for high productivity. This review systematically summarizes the major pharmacological properties of PCA, including its antioxidant, anti-inflammatory, antimicrobial, antiviral, anti-aging, neuroprotective, and hepatoprotective activities. It further highlights the therapeutic potential of PCA in the prevention and management of various chronic diseases, such as cancer, diabetes, Alzheimer’s disease, and hypertension. Furthermore, this review summarizes the representative microbial biosynthetic pathways for PCA and discusses recent progress in metabolic engineering strategies aimed at enhancing its microbial production. These strategies include reinforcing precursor supply, redirecting metabolic flux toward the shikimate pathway, blocking PCA degradation routes, relieving intracellular feedback regulation, improving host tolerance, and optimizing fermentation processes to achieve higher PCA productivity and yield. Finally, the major bottlenecks limiting PCA biomanufacturing are discussed, and prospective directions for future research are proposed.

## 1. Introduction

**Figure 1:**
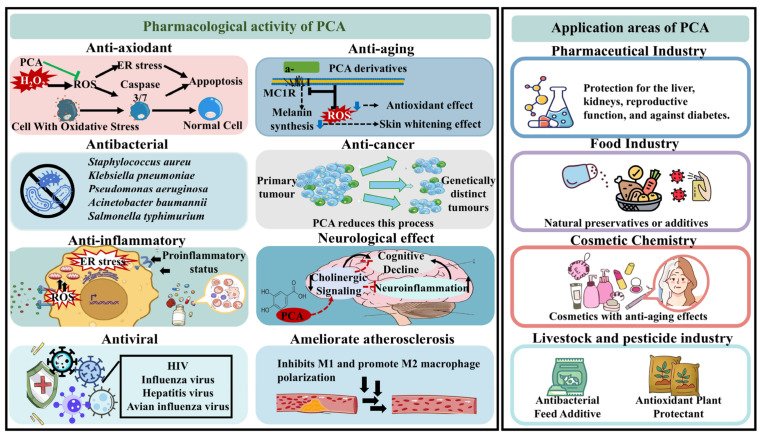
Pharmacological activities of PCA and its applications in pharmaceuticals, healthcare, food, cosmetics, livestock and pesticide industries.

Protocatechuic acid (3,4-Dihydroxybenzoate, PCA) is a naturally occurring phenolic acid widely found in vegetables, fruits, and medicinal plants. Despite its simple chemical structure, PCA exhibits remarkable biological versatility and functions as an important metabolic intermediate in the metabolism of anthocyanins and proanthocyanidins [1]. As a natural polyphenolic compound, PCA exhibits remarkable pharmacological activities, including antioxidant [2,3], antimicrobial [4,5], anti-inflammatory [6,7], antiviral [8], anti-aging [9], anticancer [10], and neuroprotective effects [11], conferring broad applicability across the pharmaceutical, functional food, cosmetic, livestock, and agrochemical sectors [12] (Figure 1). Industrially, PCA and its copolymers have been employed as high-potential electrochemical electrode materials, and PCA also serves as a pivotal precursor for high-value aromatic compounds such as vanillin and vanillic acid [13]. Microbial biosynthetic pathways for PCA enable the production of high-value intermediates, including 4-hydroxybenzoate (4-HBA), vanillin, and 3,4-dihydroxymandelate. In the biomedical field, PCA exerts protective effects on the liver, kidney, and reproductive system, and demonstrates potential in the prevention and treatment of chronic diseases, including diabetes, Alzheimer’s disease, osteoporosis, nonalcoholic fatty liver disease, atherosclerosis, and hypertension [14,15,16]. Notably, PCA displays concentration-dependent pharmacological activities, with high concentrations inhibiting cell proliferation and inducing apoptosis, while low concentrations can promote cell growth, underscoring the importance of precise dose control in clinical applications and functional product development [14]. In the food industry, PCA serves as a natural preservative or functional additive and can be embedded in hydrogel-based matrices or microencapsulation systems to prolong shelf life and enhance physicochemical stability and sensory quality [12]. In cosmetics, PCA and its derivatives promote dermal fibroblast differentiation and type I collagen synthesis, thereby exerting anti-wrinkle and anti-aging effects [9]. In livestock and agriculture, PCA displays broad-spectrum antimicrobial and antioxidant activities, serving as an effective alternative to antibiotic growth promoters to enhance gut health, nutrient absorption, and production performance, while mitigating residue, resistance, and toxicity concerns [17]. Currently, with the steadily increasing global demand for PCA, extraction using organic solvents from the pigmented scales of selected onion varieties remains the most widely adopted and effective production strategy [18,19]. However, from a techno-economic perspective, plant extraction is limited by the high costs associated with raw material collection, seasonal supply fluctuations, solvent consumption, labor-intensive extraction procedures, and downstream purification, all of which contribute to low productivity and poor resource utilization. Although chemical synthesis can provide relatively stable production, its dependence on petrochemical feedstocks, harsh reaction conditions, and waste treatment requirements increases both environmental impact and operating costs. In comparison, microbial fermentation has emerged as a promising alternative by utilizing inexpensive renewable feedstocks under mild operating conditions, thereby reducing raw material dependence while offering advantages in process scalability, product consistency, and long-term economic competitiveness. Nevertheless, further improvements in metabolic engineering, fermentation performance, and process integration are still required to achieve industrially competitive production costs and support the commercial manufacture of PCA [20].

Although research on PCA has expanded rapidly in recent years, a comprehensive review systematically integrating its biological activities with emerging advances in microbial biosynthesis and metabolic engineering remains unavailable. This review aims to provide a comprehensive overview of PCA by summarizing its pharmacological functions and applications, comparing microbial biosynthetic pathways and metabolic engineering strategies in representative hosts, including *Corynebacterium glutamicum*, *Escherichia coli*, and *Pseudomonas putida*, and critically evaluating the key challenges limiting high-level PCA production, such as precursor availability, carbon flux imbalance, cofactor constraints, product toxicity, and pathway regulation. Furthermore, emerging strategies for improving microbial cell factory performance are discussed, together with future research perspectives for developing sustainable, high-efficiency, and industrially competitive microbial platforms for PCA production.

To ensure the comprehensiveness, accuracy, and transparency of this review, the literature was systematically collected from major scientific databases, including PubMed and Google Scholar. The primary focus was placed on publications published between 2015 and 2026, while seminal earlier studies were also included to provide historical context. Literature retrieval was performed using combinations of keywords including “protocatechuic acid”, “3,4-dihydroxybenzoic acid”, “biosynthesis”, “microbial production”, “*C. glutamicum* metabolic engineering”, “synthetic biology”, “shikimate pathway”, “PCA biological activity”, “ PCA antioxidant”, “ PCA anti-inflammatory”, and “ PCA neuroprotection”. Priority was given to original experimental studies, highly cited publications, and recent reports supported by robust experimental evidence, while representative review articles were consulted to identify landmark studies and emerging research trends.

## 2. Pharmacological Activities of PCA

### 2.1. Antioxidant Activity

PCA exhibits pronounced antioxidant activity both in vitro and in vivo, with its in vitro antioxidant capacity approximately tenfold higher than that of α-tocopherol [21]. Mechanistic studies indicate that PCA exerts its effects by enhancing endogenous antioxidant defense systems, suppressing inflammatory responses, and regulating apoptotic pathways, thereby conferring protective effects across multiple tissues and organs, including the heart, liver, kidney, gastrointestinal tract, hypothalamus, and testes [22,23]. Both in vitro and in vivo studies show that PCA attenuates palmitic acid-induced oxidative damage in human umbilical vein endothelial cells and reduces aortic oxidative stress in high-fat diet-fed mice, highlighting its potential for vascular protection under metabolic stress [24,25]. Moreover, PCA can modulate the miR-219a-5p/p66Shc signaling axis, upregulating miR-219a-5p and downregulating p66Shc, thereby alleviating alcohol-induced hepatic injury [26]. In the nervous system, PCA mitigates cadmium- and arsenic-induced cortical neurotoxicity by suppressing oxidative stress, neuroinflammation, and apoptosis-related pathways, demonstrating neuroprotective potential [22,23]. Mechanistically, PCA enhances glutathione peroxidase and superoxide dismutase activities, inhibits xanthine oxidase and NADPH oxidase activities, and reduces malondialdehyde levels, collectively attenuating systemic oxidative stress [27]. PCA also functions as an efficient peroxyl radical scavenger in polar aqueous environments and retains free radical-scavenging activity in nonpolar lipid environments, indicating adaptive antioxidant potential across physiological microenvironments [28]. Recent advances in biomaterials have enabled the grafting of PCA onto chitosan or carboxymethyl chitosan to create functionalized hydrogels with excellent antioxidant performance and biocompatibility, allowing sustained release of PCA monomers and providing new strategies for antioxidant drug delivery and tissue engineering applications. Overall, PCA exerts both direct radical-scavenging effects and multi-targeted modulation of oxidative stress-related signaling pathways, though current evidence is primarily derived from cellular and animal models, and comprehensive evaluation of its pharmacokinetics, long-term safety, and clinical translation remains necessary.

### 2.2. Antimicrobial Activity

PCA demonstrates broad-spectrum antimicrobial activity against various microorganisms, with inhibitory potency ranked as follows: *Staphylococcus aureus* > *Aspergillus flavus* > *Lactobacillus monocytogenes* > *E*. *coli* > *Bacillus cereus* > *Pseudomonas aeruginosa* [4,5]. In vitro studies show that PCA effectively inhibits the growth of clinically relevant pathogens such as *S. aureus*, *Klebsiella pneumoniae*, *P. aeruginosa*, and *Acinetobacter baumannii*, as well as food spoilage organisms including *Salmonella typhimurium* DT104, *E. coli* O157:H7, *Listeria monocytogenes* [29,30]. These findings indicate that PCA is not strain-specific but exhibits activity against a range of microorganisms. Its antimicrobial mechanisms involve disruption of cell membrane integrity and interference with metabolic processes. For example, the inhibitory activity of PCA against *M. luteus* has been associated with these mechanisms, suggesting a multi-target mode of action that may reduce the likelihood of resistance development [31]. In food and feed applications, PCA serves as a natural preservative or functional additive by preventing pathogen contamination and mycotoxin formation while selectively sparing beneficial microorganisms, thereby balancing food safety with the maintenance of gut microbiota integrity [32]. Despite these promising in vitro findings, studies investigating the stability of PCA in complex food matrices and in vivo systems, optimal dosage, long-term safety, and the underlying molecular mechanisms remain limited.

### 2.3. Anti-Inflammatory and Analgesic Activity

PCA exerts anti-inflammatory effects via multi-target mechanisms involving inflammatory signaling pathways, oxidative stress modulation, and the aryl hydrocarbon receptor axis [33]. In vitro, PCA reduces the production of inflammatory mediators in LPS-stimulated BV2 microglial cells and keratinocytes, whereas in vivo studies have demonstrated its therapeutic efficacy in murine models of colitis and osteoarthritis [34]. In chronic obstructive pulmonary disease models, PCA alleviates pathological symptoms, supporting its potential for the treatment of diverse inflammatory disorders [35]. However, its low bioavailability restricts its therapeutic efficacy and limits its clinical applicability [36]. Novel delivery systems, such as dextran-coated iron oxide nanoparticles, have been developed to enhance PCA cellular uptake and enable magnetic targeting, while also providing theragnostic capabilities and potentiating its anti-inflammatory effects [37]. Furthermore, PCA can cross the blood–brain barrier and exert central analgesic effects, showing time- and dose-dependent efficacy in murine hot-plate and tail-immersion tests, while indirectly suppressing peripheral pain. Its analgesic mechanism is thought to involve the spinal cannabinoid system, although the precise molecular basis remains to be fully elucidated [38,39]. Collectively, these studies highlight the promising potential of PCA in both inflammation modulation and central pain management.

### 2.4. Antiviral Activity

PCA demonstrates broad-spectrum antiviral activity against HIV, influenza viruses, hepatitis viruses, and avian influenza viruses [40,41]. In vitro, PCA inhibits HSV-2 replication more effectively than acyclovir while exhibiting lower cytotoxicity, suggesting potential clinical utility [42]. Animal studies confirm its antiviral efficacy, showing protection against H9N2 and H1N1 influenza virus infections in mice and reduction in pulmonary inflammation via modulation of the TLR4/NF-κB pathway [43]. Additionally, PCA enhances survival in chickens infected with infectious bursal disease virus and prevents avian influenza virus infection in mice, highlighting potential applications in poultry viral disease prevention [44]. These findings indicate that PCA may act through multiple mechanisms, including direct inhibition of viral replication and modulation of host immune signaling, while exhibiting a favorable safety profile and low cytotoxicity, thereby offering a potential strategy to mitigate antiviral resistance.

### 2.5. Anti-Aging Activity

PCA exerts anti-aging and skin-protective effects through its antioxidant and photoprotective properties [45]. It absorbs UVB, restores redox balance in vitro, enhances cell viability, mitigates UVB-induced oxidative damage, scavenges free radicals, promotes type I collagen synthesis, and inhibits UV-induced MMP-1 expression, collectively contributing to anti-wrinkle and photoprotective effects [45]. Previous studies suggest that PCA may promote human dermal fibroblast differentiation and type I collagen synthesis in skin explants, further supporting its structural benefits to the skin [9]. In vivo, PCA enhances antioxidant capacity in the spleen and liver of aged rats, indicating systemic anti-aging and organ-protective potential [46]. Nevertheless, most available data are derived from in vitro or animal models, with limited evaluation of long-term effects, dose dependency, and metabolism in human skin. Bioavailability and dermal penetration remain key challenges for clinical translation.

### 2.6. Anticancer Activity

PCA exhibits anti-proliferative and pro-apoptotic effects across multiple cancer cell types, including breast, gastric, liver, osteosarcoma, leukemia, oral, and colorectal cancers, demonstrating multi-target anticancer potential [47]. Mechanistically, PCA modulates redox homeostasis, inhibits the HO-1 system, activates p21-mediated apoptotic pathways, suppresses epithelial–mesenchymal transition, and blocks the PI3K/Akt/mTOR signaling axis [47]. In glioma models, PCA regulates the NLRP3/Caspase-1/GSDMD pathway to inhibit proliferation, migration, and pyroptosis in a dose-dependent manner (*p* < 0.05), indicating potential in neuro-oncology. Nanocarrier-based delivery of PCA (e.g., graphene, graphene oxide–PEG, ZnAl materials) enhances anticancer activity by improving stability, bioavailability, sustained release, and targeted delivery, resulting in stronger inhibition of HEP-G2 hepatocellular carcinoma and HCT116 colorectal cancer cells, as well as mouse liver cancer models, highlighting nanotechnology’s potential to augment PCA’s therapeutic efficacy [48,49].

### 2.7. Neuroprotective Activity

PCA exerts neuroprotective effects through antioxidant, anti-inflammatory, and anti-apoptotic mechanisms and has attracted considerable interest as a potential natural neuroprotective agent for Alzheimer’s disease, Parkinson’s disease, and hypoxic–ischemic brain injury [50,51]. PCA enhances PC12 cell viability, reverses H_2_O_2_-induced apoptosis, and increases intracellular glutathione and catalase activity, thereby mitigating oxidative stress-induced neuronal damage [52]. In Alzheimer’s models, PCA reduces Aβ plaque deposition and tau hyperphosphorylation, prevents metal ion dysregulation and reactive oxygen species overproduction, and improves cognitive function [53]. Behavioral studies have confirmed improvements in learning and memory, as well as enhanced neuronal regeneration. Combined administration with baicalin synergistically enhances the protection of substantia nigra dopaminergic neurons in Parkinson’s disease models, improving motor deficits without inducing cardiac side effects [54]. In hypoxic–ischemic brain injury, PCA modulates the HIF-1α/VEGFA axis to inhibit ferroptosis, ameliorating neuronal microenvironment and reducing damage [55]. While PCA has shown robust neuroprotective effects across multiple experimental models and pathological pathways, including oxidative stress, inflammation, metal ion homeostasis, and programmed cell death, most of the available evidence is still preclinical in nature. In addition, pharmacokinetic and clinical data are still limited, highlighting the need for further multi-omics analyses and disease-model studies to elucidate its mechanisms and assess its translational potential.

### 2.8. Anti-Atherosclerotic Effects

PCA exhibits significant multi-target potential in preventing and attenuating atherosclerosis, a chronic inflammatory and proliferative vascular disease involving endothelial dysfunction, inflammation, vascular smooth muscle cell proliferation, and extracellular matrix remodeling [54,56]. Oxidized low-density lipoprotein (oxLDL) is a key pathogenic factor, promoting atherogenesis via endothelial injury and p53-dependent macrophage apoptosis. PCA protects vascular endothelial cells from oxLDL-induced damage, inhibits oxLDL-mediated p53-dependent macrophage apoptosis, suppresses pro-inflammatory M1 macrophage polarization via the PI3K/Akt/NF-κB/SOCS1 pathway, and enhances anti-inflammatory M2 polarization through the STAT6/PPARγ pathway, thereby slowing disease progression in high-cholesterol diet-fed mice [57]. Additionally, PCA inhibits vascular smooth muscle cell proliferation and regulates lipid metabolism through PPARγ activation, reducing lipid uptake and synthesis, ameliorating non-alcoholic fatty liver, and mitigating systemic lipid metabolic disturbances that exacerbate atherosclerosis [58]. Collectively, these findings demonstrate PCA’s multi-target, systemic cardioprotective effects, providing a mechanistic rationale for its potential as a natural cardiovascular-protective agent.

### 2.9. Pharmacokinetics and Bioavailability of PCA

Despite the diverse pharmacological activities of protocatechuic acid (PCA), its clinical translation remains limited by the relatively small amount of pharmacokinetic and bioavailability data available in humans [59]. Following oral administration, PCA is rapidly absorbed and eliminated, with glucuronidation and sulfation representing the predominant metabolic pathways. In the first dedicated human pharmacokinetic study, healthy volunteers consumed chicory containing naturally occurring PCA, and free PCA reached a maximum plasma concentration of 3273 ± 729 nmol/L at approximately 1 h, whereas glucuronide and sulfate conjugates peaked at 4 h [60]. The elimination half-life of free PCA was approximately 1.7 h, indicating rapid systemic clearance. Furthermore, approximately 23.8% of the ingested PCA was recovered in the circulation, while 12.2% and 12.8% were recovered in urine and feces, respectively, demonstrating that PCA is bioavailable but undergoes extensive phase II metabolism after absorption [60]. Importantly, glucuronide and sulfate conjugates accounted for the majority of PCA recovered in biological samples, suggesting that these metabolites may contribute substantially to its biological effects.

In addition to direct dietary intake, PCA is an important microbial metabolite of anthocyanins, proanthocyanidins, and other complex polyphenols, indicating that gut microbiota plays a crucial role in determining systemic PCA exposure. Current evidence suggests that rapid metabolism and elimination may limit the bioavailability of free PCA and consequently reduce its therapeutic efficacy. To overcome these limitations, various delivery strategies, including nanoparticle-based formulations, liposomes, polymeric carriers, and cyclodextrin inclusion complexes, have been explored in preclinical studies to enhance solubility, improve stability, prolong circulation time, and increase tissue distribution [61]. However, these approaches remain largely experimental, and systematic pharmacokinetic evaluations, including dose proportionality, tissue distribution, metabolite profiling, and long-term safety, are still lacking. More importantly, no PCA-based therapeutic agent has yet progressed to well-designed clinical efficacy trials, leaving a substantial gap between encouraging preclinical findings and clinical application [59]. Future studies should integrate optimized drug delivery systems with comprehensive pharmacokinetic analyses and randomized clinical studies to establish exposure–response relationships and accelerate the clinical translation of PCA. Currently, PCA is mainly utilized as a pharmaceutical intermediate or chemical precursor for the synthesis of bioactive compounds rather than as an independent therapeutic molecule. Therefore, further investigations into human pharmacokinetics, safety profiles, optimal dosage, and well-designed clinical trials are required to facilitate the translational development of PCA.

## 3. Biosynthesis and Engineering Strategies for PCA Production

The traditional plant-based extraction of PCA is intrinsically constrained by low yield, labor-intensive and time-consuming processing, and suboptimal utilization of biomass, frequently accompanied by environmental burdens [62,63]. As a result, this approach is insufficient to satisfy the rapidly growing market demand for PCA. In response, recent studies have increasingly explored alternative biosynthetic approaches, among which microbial fermentation has emerged as a particularly promising and scalable platform [64]. In contrast to plant-based extraction, microbial fermentation confers several intrinsic advantages, including precise process controllability, abbreviated production cycles, and scalability suitable for industrial implementation. Coupled with recent advances in metabolic engineering, this strategy has demonstrated considerable potential for high-titer PCA biosynthesis. For example, metabolically engineered *E. coli* strains can efficiently convert 3-dehydroshikimate (DHS) (via the dehydratase pathway encoded by *aroZ* or *qsuB*) into PCA, achieving reported titers of up to 46.65 g/L with a glucose conversion yield of approximately 0.23 g/g [65]. Microbial fermentation offers a sustainable and environmentally friendly platform for PCA production. In this system, inexpensive and renewable carbon sources such as glucose are converted into PCA through engineered biosynthetic pathways, which are optimized to efficiently direct metabolic flux toward the desired product.

### 3.1. Chassis Microorganisms for Microbial Production of PCA

Microbial hosts currently employed for PCA biosynthesis encompass *Bacillus thuringiensis* [66], *E. coli* [65,67,68], *Bacillus anthracis* [69], *C*. *glutamicum* [70,71,72], *Bacillus licheniformis* [73], and *P*. *putida* [74,75] (Table 1). These organisms exhibit pronounced differences in metabolic network, carbon flux allocation, and tolerance to aromatic compounds, offering a spectrum of options for the rational design and optimization of PCA biosynthetic systems. Such host diversity not only expands the engineering design space but also underpins the development of tailored strategies to enhance PCA production. Among these microorganisms, *E. coli*, *C. glutamicum*, and *P. putida* have emerged as the most extensively characterized and representative chassis for PCA biosynthesis (Table 1). *E. coli* is widely used as a microbial host owing to its well-established genetic manipulation tools, extensive metabolic engineering toolkit, and strong capacity for high-level production [20,65,76]. Nevertheless, excessive intracellular accumulation of PCA can intensify cytotoxic effects, leading to growth inhibition and thereby limiting further improvements in production yield. This challenge necessitates the development of mitigation strategies, including membrane engineering, adaptive laboratory evolution (ALE), and transporter engineering/optimization. In contrast, *C. glutamicum* represents a promising industrial host owing to its biosafety status, rapid growth, and robust aromatic metabolism. However, endogenous PCA degradation pathways can divert carbon flux away from product accumulation [70]. *P. putida* exhibits strong tolerance to oxidative stress and high concentrations of phenolic compounds, while its unique central carbon metabolism generates abundant reducing power, which is highly favorable for aromatic compound biosynthesis [74]. Despite these intrinsic merits, its practical application is often limited by the requirements for extensive genetic modification and suboptimal transmembrane transport capacity.

Collectively, although each chassis presents distinct advantages, *E. coli* in production capacity, *C. glutamicum* in industrial safety and robustness, and *P. putida* in stress resilience, they share common challenges in microbial PCA production. PCA cytotoxicity compromises membrane integrity and imposes a significant metabolic burden, thereby inhibiting cell proliferation [82]. Concurrently, competition for carbon flux within aromatic metabolic networks, together with endogenous PCA degradation pathways, reduces overall PCA yield, highlighting the need for systematic metabolic network rewiring to minimize carbon loss. Achieving an optimal balance among increased production capacity, precise carbon flux distribution, improved cellular tolerance, and effective suppression of competing pathways remains a central challenge in microbial PCA biosynthesis. Before metabolic engineering interventions, a comprehensive understanding of the biosynthetic pathway and their regulatory mechanisms is indispensable for improving microbial PCA production efficiency.

### 3.2. Microbial Biosynthetic Pathways for PCA

**Figure 2:**
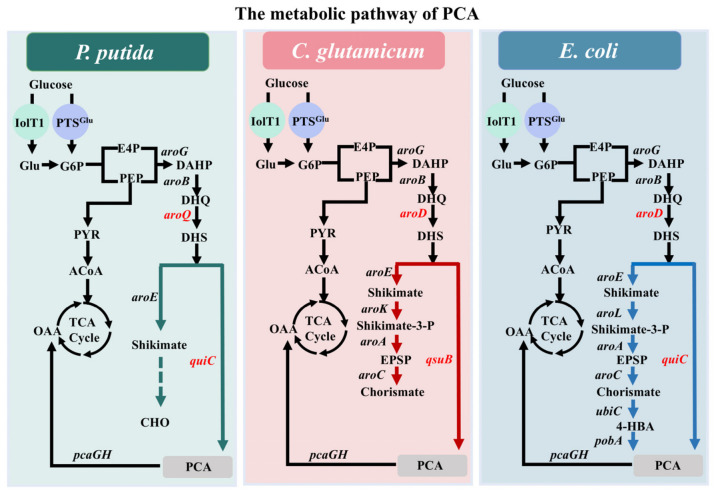
The biosynthesis pathway of PCA in *P. putida*, *C. glutamicum*, and *E. coli*. *aroG*, encodes DAHP synthase; *aroB*, encodes DHQ synthase; *aroQ*/*aroD*, encodes DHQ dehydratase; *aroE*, encodes shikimate dehydrogenase; *aroK*/*aroL*, encodes shikimate kinase; *aroA*, encodes EPSP synthase; *aroC*, encodes chorismate synthase; *quiC*/*qsuB*, encodes DHS dehydratase; *ubiC*, encodes chorismate pyruvate lyase; *pobA*, encodes 4-HBA 3-hydroxylase; *pcaHG*, encodes PCA 3,4-dioxygenase; *iolT1*, encodes myo-inositol permease; *ptsGHI*, encodes glucose specific sugar:phosphoenolpyruvate phosphotransferase; Glu, glucose; G6P, glucose-6-phosphate; PEP, phosphoenolpyruvate; E4P, erythrose 4-phosphate; PYR, pyruvate; ACoA, acetyl coenzyme; DAHP, 3-deoxy-D-arabinoheptulosonate-7-phosphate; DHQ, 3-dehydroquinate; DHS, 3-dehydroshikimate; EPSP, 5-enolpyruvoylshikimate 3-phosphate; 4-HBA,4-hydroxybenzoate; OAA, oxaloacetate; PCA, protocatechuic acid.

In microorganisms, PCA biosynthesis primarily relies on the DHS pathway, which comprises two core routes: the direct DHS dehydration pathway and the branched 4-HBA derivative pathway (Figure 2). Glucose is initially metabolized through the Embden–Meyerhof–Parnas (EMP) and pentose phosphate pathways (PPP) to generate phosphoenolpyruvate (PEP) and erythrose-4-phosphate (E4P), which are condensed by DAHP synthase (encoded by *aroG*) to form 3-deoxy-D-arabino-heptulosonate-7-phosphate (DAHP) (Figure 2). DAHP is then converted to 3-dehydroquinate (DHQ) by DHQ synthase (encoded by *aroB*) and subsequently dehydrated to DHS via DHQ dehydratases (encoded by *aroQ* and *aroD*) (Figure 2). In the direct DHS dehydration pathway, DHS dehydratase (encoded by *qsuB* or *aroZ*) catalyzes the direct conversion of DHS into PCA [83] (Figure 2). This pathway naturally exists in chassis organisms such as *C. glutamicum* and can be heterologously expressed in microbes like *E. coli* to achieve efficient PCA production [84]. In the branched 4-HBA derivative pathway, DHS is initially reduced to shikimate by AroE, followed by its conversion to chorismate via AroC and AroA. Chorismate is subsequently cleaved by heterologously expressed chorismate pyruvate-lyase to yield 4-HBA, which is ultimately hydroxylated to PCA by 4-HBA 3-hydroxylase [85] (Figure 2).

**Figure 3:**
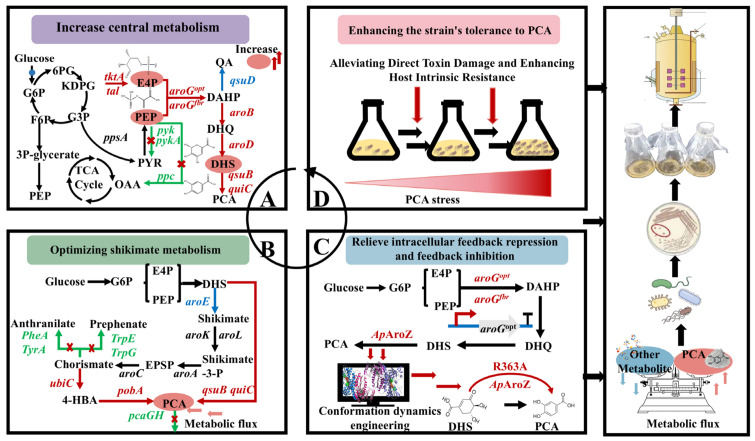
Metabolic engineering strategies for improving PCA production. A. Rewiring of central carbon metabolism. B. Engineering of the shikimate pathway. C. Alleviation of intracellular feedback regulation. D. Enhancement of cellular tolerance to PCA.

The two biosynthetic routes exhibit marked differences in host compatibility, production yield, and amenability to process optimization. The direct DHS dehydration pathway generally affords superior carbon conversion efficiency and higher PCA titers. In contrast, the 4-HBA–derived pathway is often constrained by the cytotoxicity of the intermediate 4-HBA, as well as limitations associated with rate-limiting enzymatic steps, collectively leading to comparatively lower PCA production performance [85]. Moreover, the efficiency of the shikimate pathway is intrinsically constrained by multiple factors, including the limited availability of precursors PEP and E4P carbon flux partitioning through the phosphotransferase system (PTS) [86], and stringent feedback inhibition of DAHP synthase by aromatic amino acids. To overcome these limitations, various metabolic engineering strategies have been developed, including reinforcement of central metabolic pathway, manipulating shikimate pathway, blocking degradative pathways, alleviating feedback inhibition, and enhancement of host tolerance to PCA (Figure 3). Collectively, these strategies facilitate systematic reprogramming of metabolic flux toward PCA biosynthesis, thereby underpinning high-titer, robust, and sustainable microbial production.

### 3.3. Metabolic Engineering Strategies for the Development of PCA-Producing Strains

#### 3.3.1. Reprogramming Central Metabolism to Enhance Flux Toward PCA Biosynthesis

**Figure 4:**
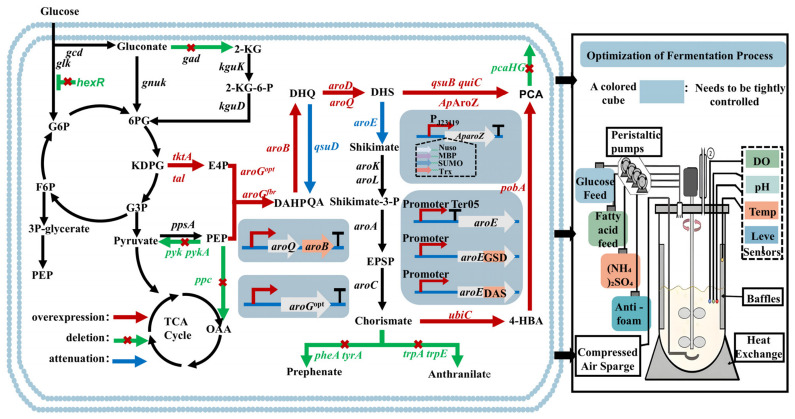
Gene modulation and fermentation optimization for the development of PCA producing strains. *gcd*, encodes glucose dehydrogenase; *gad*, encodes gluconate 2-dehydrogenase; *glk*, encodes glucokinase; *gnuk*, encodes gluconokinase; *edd*, encodes phosphogluconate dehydratase; *eda*, encodes 2-keto-3-deoxy-6-phosphogluconate aldolase; *kguK*, encodes 2-ketogluconate kinase; *kguD*, encodes 2-ketogluconate-6-P reductase; *tktA*/*tal*, encodes KDPG aldolase; *ppc*, encodes pyruvate carboxylase; *ppsA*, encodes phosphoenolpyruvate synthase; *pckA*/*pyk*, encodes phosphoenolpyruvate carboxykinase; *aroG*^opt^/*aroG*^fbr^, encodes DAHP synthase; *pheA*, encodes chorismate mutase/prephenate dehydratase; *tyrA*, encodes chorismate mutase/prephenate dehydrogenase; *trpE*, encodes anthranilate synthase subunit; *ApAroZ*, 3-dehydroshikimate dehydratase. 2-KG, 2-ketogluconate; 2-KG-6-P, 2-ketogluconate-6-P; 6PG, 6-phosphogluconate; KDPG, 2-keto-3-deoxy-6-phosphogluconate; F6P, fructose 6-Phosphate; G3P, glyceraldehyde 3-Phosphate; QA, quinic acid; Phe, phenylalanine; Tyr, tyrosine; Trp, tryptophan.

The biosynthesis of PCA is initiated from two pivotal sugar-derived precursors, E4P and PEP, which serve as the fundamental building blocks for aromatic compound formation [87,88] (Figure 3). Adequate supply of these precursors is therefore essential for achieving high-level PCA production. However, in native microbial hosts, precursor availability is often constrained by the inherent distribution of carbon flux, limiting the efficiency of PCA biosynthesis. For example, in *P. putida* KT2440, glucose is predominantly metabolized via the oxidative pathway, leading to the accumulation of 2-ketogluconate and consequent carbon loss [89]. In contrast, targeted deletion of glucose dehydrogenase can effectively redirect carbon flux toward PEP and E4P, thereby enhancing PCA biosynthesis without markedly compromising cell growth, underscoring the importance of rational carbon flux reallocation [81]. Accordingly, strategies to enhance precursor availability can be broadly classified into two categories. The first focuses on directly strengthening core precursor biosynthetic pathways. For instance, overexpression of *ppsA* under an IPTG-inducible promoter can increase PEP supply, while amplification of *tktA* enhances flux through the PPP, thereby elevating E4P generation and improving NADPH availability [80] (Figure 4). The second strategy focuses on systematically reducing competing carbon fluxes by modulating genes such as *ppc*, *pyk*, and *pykA*, thereby limiting the conversion of PEP to oxaloacetate and redirecting carbon flow toward the shikimate pathway [89] (Figure 4). Notably, *pykA* has been shown to exert a stronger influence on precursor accumulation than *pyk*, underscoring the necessity of fine-tuning glycolytic flux for efficient PCA biosynthesis. Collectively, systematic optimization of central carbon metabolism, through coordinated enhancement of precursor supply and suppression of competing fluxes, increases the availability of key intermediates. This integrated strategy ultimately maximizes carbon flux toward PCA production.

#### 3.3.2. Alleviating Feedback Inhibition to Promote PCA Biosynthesis

In PCA biosynthesis, a major metabolic bottleneck occurs at the condensation of PEP and E4P to form DAHP, a reaction catalyzed by DAHP synthase. This reaction is subject to strong feedback inhibition by aromatic amino acids, which significantly constrains precursor availability and limits downstream PCA biosynthesis [90]. To address this bottleneck, researchers have engineered feedback-resistant DAHP synthase variants through targeted 148-base mutations and implemented these modifications in conjunction with *hexR* deletion in *E. coli* or *P. putida* KT2440, leading to pronounced accumulation of DAHP [91,92]. For example, Li et al. demonstrated that P*_tac_*-driven expression of *aroG^fbr^* coupled with *hexR* knockout increased PCA titer to 338.5 mg/L after 72 h of shake-flask fermentation, a 3.7-fold improvement over the control [74]. This highlights the critical role of feedback alleviation in enhancing metabolic flux. In addition, PCA exerts competitive inhibition on 3-dehydroshikimate dehydratase (AroZ) (Figure 3). Protein conformational engineering strategies, such as identifying residue R363A and constructing the ApAroZ variant, disrupted intra-enzyme salt bridges to favor an “open” conformation, increasing the product inhibition constant by 61.33% and catalytic efficiency by 19.78%, ultimately achieving 37.02 g/L PCA in a 5 L bioreactor [65]. These results demonstrate that combining feedback-resistant DAHP synthase with structure-guided enzyme engineering can simultaneously relieve precursor limitation, providing an efficient approach for improving microbial PCA production (Figure 3). Alleviating feedback regulation of AroG not only significantly enhances flux and precursor accumulation but also exemplifies a modern metabolic engineering paradigm that integrates pathway flux optimization with enzyme structural modulation.

#### 3.3.3. Enhancing PCA Biosynthesis Through Manipulating Shikimate Pathway

The condensation of E4P and PEP channels carbon flux into the shikimate pathway, generating DHS as a key intermediate, which subsequently leads to PCA biosynthesis. Accordingly, targeted metabolic engineering of the shikimate pathway represents a central strategy for improving PCA production (Figure 3). Notably, previous studies have shown that co-enhancing the native DHS pathway alongside a heterologous 4-HBA pathway leads to a synergistic improvement in PCA production, with the endogenous DHS route in *C. glutamicum* playing a major role in driving PCA accumulation [80]. In *C. glutamicum*, construction of a dual-pathway system integrating the DHS and 4-HBA routes led to the development of the PCA03 strain, which achieved a PCA titer of 41.7 g/L, thereby demonstrating that pathway synergy effectively alleviates the metabolic bottlenecks inherent in single-route systems [80]. Further optimization of PCA production primarily relies on precise redistribution of carbon flux, particularly by enhancing the conversion of DHS toward PCA while minimizing flux through competing pathways. A representative strategy involves modulating the expression of key enzymes to rebalance metabolic flux. For example, in *P. putida* KT2440, insertion of the terminator Ter05 in the downstream region of *aroE* partially downregulated its expression, thereby redirecting carbon flux toward DHS and yielding the PCA15 strain with a titer of 11.0 g/L, corresponding to a 111.5% increase over the control [74] (Figure 4). Subsequent co-enhancement of *qsuB* expression and partial attenuation of *aroE* further increased PCA production while preserving aromatic amino acid biosynthesis, emphasizing the importance of balanced metabolic control. Beyond transcriptional regulation, post-translational approaches, including the introduction of protein degradation tags, have been used to dynamically modulate AroE levels, allowing finer metabolic flux control while maintaining cellular robustness [81]. Moreover, reinforcement of heterologous pathways represents an effective strategy for enhancing PCA biosynthesis. For example, expression of UbiC from *E. coli* in *C. glutamicum*, as well as co-expression of DSD combined with the deletion of competing pathway genes (*pheA*, *tyrA*, and *trpE*) in *E. coli* ATCC 31882, significantly increased PCA production [77] (Figure 4). To mitigate the metabolic burden imposed by the implementation of complex biosynthetic pathways in a single microbial host, modular co-culture strategies have been proposed. By partitioning the PCA biosynthetic pathway into upstream (glucose to 4-HBA) and downstream (4-HBA to PCA) modules, and co-culturing engineered strains such as UY3R and HAYT at an optimized ratio (9:1), significantly improved titer and productivity were achieved relative to monoculture systems, underscoring the potential of modular co-culture design for high-efficient PCA production [78]. Collectively, these strategies, encompassing carbon flux redistribution, dynamic enzyme regulation, heterologous pathway engineering, and modular design, enable systematic optimization of the shikimate pathway, thereby promoting efficient PCA accumulation.

#### 3.3.4. Enhancing PCA Accumulation by Blocking Degradative Pathways

During microbial production of PCA, the compound can be further catabolized by the host mainly via the classical β-ketoadipate pathway, through which PCA is converted into acetyl-CoA and succinyl-CoA for entry into the tricarboxylic acid (TCA) cycle [93]. This process redirects carbon back into central metabolism, thereby reducing PCA accumulation. Accordingly, targeted disruption of PCA degradative pathways to minimize product reutilization has emerged as a key metabolic engineering strategy for improving PCA accumulation. The *pcaGH* gene cluster, encoding the protocatechuate dioxygenase complex, plays a key role in this degradation pathway (Figure 4). Its deletion effectively blocks the conversion of PCA into downstream metabolites, thereby preventing its entry into central metabolism. However, deletion of *pcaGH* alone is generally insufficient to achieve substantial PCA accumulation, as significant improvements typically rely on the concurrent enhancement of upstream biosynthetic flux [80]. For instance, by deleting *pcaGH* in *P. putida* to prevent PCA catabolism into the TCA cycle, while simultaneously overexpressing *quiC* to reinforce the DHS biosynthetic pathway, PCA production titer was successfully increased to 91.5 mg/L [74]. More recently, this strategy was further validated and expanded through the construction of engineered strains lacking endogenous PCA degradation pathways [79]. Fermentation analyses revealed continuous PCA accumulation throughout the cultivation process, with the PCA titer at 48 h showing a 13.48% increase relative to that at 24 h and ultimately reaching 12.48 g/L [79]. These findings highlight that alleviating endogenous product degradation constitutes an effective strategy for improving PCA production, while further emphasizing that product reutilization and metabolic competition represent major bottlenecks constraining microbial PCA biosynthesis. Notably, the β-ketoadipate pathway functions not only in the catabolism of aromatic compounds but also contributes substantially to cellular carbon balance, metabolic flexibility, and environmental adaptability in certain microorganisms. Consequently, complete disruption of this pathway may impair cell growth, compromise metabolic homeostasis, and ultimately diminish overall production performance. Therefore, future metabolic engineering efforts should adopt a more integrated and balanced design framework that simultaneously fine-tunes degradative pathways, strengthens precursor supply and carbon flux redistribution, and preserves cellular robustness, thereby enabling the coordinated achievement of high PCA titers, efficient carbon utilization, and stable host physiology.

#### 3.3.5. Enhancing Strain Tolerance to Promote PCA Accumulation

Enhancing microbial tolerance to PCA is essential for achieving high-yield production, as excessive intracellular accumulation imposes dual stresses of metabolic burden and product toxicity (Figure 3). These stresses trigger systemic cellular responses, including intensified resource competition, oxidative damage, accumulation of toxic intermediates, increased cell death, and impaired membrane integrity, collectively reducing metabolic capacity and representing a major bottleneck in industrial applications [94,95,96]. To address this challenge, strategies for improving PCA tolerance can generally be divided into two complementary approaches, namely alleviating the direct cytotoxicity of PCA and strengthening the intrinsic stress resistance of the microbial host. On the one hand, toxicity can be mitigated by reducing intracellular PCA levels or enhancing its removal, for example through in situ product removal, overexpression or optimization of major facilitator superfamily transporters to promote efflux, engineering of degradation pathways to expand detoxification networks, and modulation of global stress responses to rebalance cellular resources and maintain population homeostasis [97,98]. On the other hand, reinforcing host robustness focuses on improving the intrinsic ability of cells to withstand PCA-induced stress. This can be achieved through ALE to select high-resistance mutants or by using inherently robust chassis strains. These resistance-oriented strategies can be further enhanced by multi-omics-guided precise genome editing and membrane engineering, which improve cellular resilience at both genetic and structural levels [99]. In this context, ALE has been widely demonstrated to markedly improve tolerance to toxic metabolites. For example, Matson et al. [100] reported that an evolved strain (M20) exhibited a 2.8-fold increase in productivity under 3 g/L isobutyrate stress. Building upon conventional ALE, recent studies have further enhanced screening efficiency through the integration of biosensor-assisted selection strategies [65]. In particular, a PphcN–mKate fluorescence-based dual-selection system was developed to couple intracellular PCA accumulation with fluorescence intensity, thereby enabling real-time correlation between cellular fluorescence signals and production capacity. When combined with high-throughput fluorescence-activated cell sorting, this strategy enabled the rapid enrichment of strains exhibiting simultaneously improved PCA tolerance and biosynthetic performance. As a result, the engineered strains achieved a PCA titer of 46.65 g/L in 5 L bioreactors, with a glucose conversion yield of 0.23 g/g and a productivity of 1.46 g/L/h, effectively overcoming several key constraints under non-inducing industrial fermentation conditions [65]. These findings demonstrate that tolerance engineering not only mitigates the cytotoxic effects associated with PCA accumulation but also acts synergistically with metabolic flux optimization, thereby providing a robust and industrially scalable strategy for simultaneously improving PCA yield, productivity, and process stability.

### 3.4. Fermentation Process Optimization to Enhance PCA Accumulation

The composition and proportions of fermentation media play a critical role in microbial growth, cellular metabolic state, and the efficiency of target metabolite synthesis. Therefore, medium optimization is a key step for improving fermentation performance. In PCA production systems, medium formulation plays a decisive role in determining product yield. Building on this premise, Chung et al. [64] systematically optimized the production medium using a full factorial design, the steepest ascent method, and response surface methodology. Under the optimized conditions, the PCA titer increased to over 5 g/L in 72 h batch fermentations. This result highlights the effectiveness of statistical approaches in balancing carbon sources, nitrogen sources, and inorganic salts, thereby promoting cellular growth and directing metabolic flux toward PCA biosynthesis. Among these factors, nitrogen supply and carbon source strategy are particularly critical for PCA biosynthesis, as insufficient nitrogen limits protein synthesis and reduces the expression of key enzymes, ultimately constraining PCA production. For instance, Shi et al. [79] optimized nitrogen conditions by employing ammonium sulfate as the primary nitrogen source and adjusting the carbon-to-nitrogen ratio to 57.6, which significantly improved both biomass accumulation and PCA titer. In parallel, carbon source management governs carbon flux distribution, and, for instance, in *S. cerevisiae*, high glucose concentrations can trigger the Crabtree effect [101], diverting carbon toward ethanol and other by-products. To overcome this limitation, dynamic feeding strategies based on specific growth rate, compared with constant-rate feeding, maintain glucose levels within 20–25 g/L, thereby improving sugar conversion efficiency, enhancing biomass growth and metabolic activity, and achieving a balance between substrate utilization and PCA formation [79]. Beyond medium composition, environmental parameters, including dissolved oxygen, pH, temperature, and osmotic pressure, also play pivotal roles in shaping cellular metabolic balance and PCA synthesis. For example, staged DO control, maintaining 20–30% during the early growth phase and 10–20% during the production phase, has been shown to enhance PCA conversion efficiency, illustrating the importance of dynamic environmental regulation in fermentation optimization [79]. Furthermore, process scale-up and intensification strategies provide an additional layer of improvement in PCA yield and production stability. Two-stage fed-batch fermentation decouples cell growth from product formation by first generating high-density biomass in a complex medium and subsequently initiating biotransformation with approximately 10% (*w*/*v*) biocatalyst, achieving PCA titers up to 82.7 g/L [80]. Similarly, one-pot approaches using complementary carbon sources, such as D-glucose and D-xylose, enable simultaneous biomass accumulation and product synthesis. When combined with computational strain design, targeted metabolic engineering, and downstream separation techniques, these strategies can increase product concentration while reducing by-product formation [72]. In addition, continuous fermentation strategies alleviate PCA-associated toxicity by maintaining product concentrations below inhibitory thresholds, thereby enabling long-term stable production [64]. Notably, in a 100 L pilot-scale reactor, this approach achieved 52.77 g/L PCA, a productivity of 0.314 g/L/h, and a glucose conversion efficiency of 0.405 mol/mol over 150 h, representing one of the highest reported performances to date [79]. Taken together, these findings demonstrate that efficient microbial PCA production relies on the integration of metabolic engineering with systematic fermentation process optimization. The synergistic development of both aspects is essential for successful industrial-scale application. Accordingly, future efforts should focus on integrating intelligent dynamic control systems, real-time metabolic monitoring, and automated feeding strategies to maximize PCA yield while maintaining cellular robustness. In parallel, multi-factorial medium optimization and modular process design should be further developed to improve fermentation efficiency, reduce production costs, and facilitate industrial implementation.

## 4. Conclusions and Future Perspectives

In summary, PCA, a naturally occurring phenolic acid with diverse pharmacological activities, including antioxidant, anti-inflammatory, antitumor, and neuroprotective effects, represents a high-value bioactive compound with broad applications, particularly as a versatile precursor for pharmaceuticals and functional products. In response to the growing demand for sustainable and efficient production methods, recent advances in metabolic engineering and synthetic biology have enabled the construction of PCA biosynthetic pathways in microbial chassis such as *C. glutamicum*, *P. putida*, and *E. coli*. Within these engineered systems, production has been substantially improved through coordinated strategies, including systematic enhancement of the shikimate pathway to increase precursor availability, optimization of key metabolic intermediates, and inactivation of competing catabolic pathways, collectively directing more efficient flux toward PCA biosynthesis. Notably, engineered *C. glutamicum* strains have achieved PCA titers up to 82.7 g/L [80], highlighting the promise of microbial fermentation as a sustainable platform for green PCA production. Despite these advances, industrial-scale implementation of microbial PCA production continues to face significant challenges, including complex metabolic flux distribution, intracellular accumulation of toxic products, imbalances in cellular energy and redox supply, and limited robustness of chassis strains, which together remain major bottlenecks to large-scale commercialization. Future development of microbial PCA production is anticipated to rely on integrated advances across three interconnected levels: systemic metabolic network optimization, intelligent cell factory design, and biomanufacturing process intensification.

In terms of metabolic engineering, efforts should shift from single-gene or pathway-specific modifications to comprehensive reconstruction of global metabolic networks. By enhancing the supply of key precursors such as PEP and E4P, redirecting flux between central carbon metabolism and aromatic amino acid pathways, and implementing dynamic metabolic control strategies, it is possible to achieve growth–production phase switching at different fermentation stages, thereby improving carbon utilization efficiency and PCA synthesis flux. The advent of CRISPR/Cas-based multiplex genome editing, synthetic promoter libraries, and programmable transcriptional regulators enables precise multi-gene expression tuning, further optimizing flux through the shikimate pathway. Complementing these strategies, machine learning-assisted enzyme engineering and computer-aided strain design provide powerful tools for rational enzyme modification and metabolic network prediction, offering a more systematic and accelerated alternative to traditional empirical optimization approaches.

In terms of cell factory, research should prioritize enhancing host tolerance and product secretion mechanisms. High intracellular PCA concentrations can impose cytotoxic stress on cell membranes and disrupt metabolic homeostasis, making strategies such as ALE, membrane engineering, and global regulatory factor modification essential for constructing robust chassis capable of withstanding high PCA production. Moreover, identification and engineering of efficient PCA transporters, guided by multi-omics analyses and membrane protein discovery, could establish a high-efficiency “intracellular synthesis–extracellular accumulation” production mode. Modular metabolic pathway design and co-culture systems further offer opportunities to distribute metabolic burden and stabilize complex networks, collectively enhancing production performance and scalability.

In terms of biomanufacturing, the future of PCA production will increasingly depend on digital and intelligent fermentation technologies. By integrating online metabolic monitoring, automated substrate feeding, and digital process control, key parameters such as substrate concentration, dissolved oxygen, and intracellular metabolic states can be dynamically regulated to maintain optimal conditions for PCA synthesis. When combined with systems biology data, metabolic modeling, and artificial intelligence algorithms, these capabilities enable the establishment of closed-loop “design–build–test–learn” platforms, accelerating the development of high-performance strains. Furthermore, coupling these approaches with continuous fermentation, cell immobilization, and environmentally friendly downstream separation techniques is expected to enhance productivity while reducing production costs. In particular, future downstream processing should further incorporate green chemistry principles and advanced physical separation technologies, such as dynamic vibration membrane filtration systems, which can efficiently alleviate membrane fouling, facilitate the removal of cell debris, and improve the recovery of high-concentration PCA from high-viscosity fermentation broths. Taken together, the convergence of synthetic biology, AI-driven strain and process design, and intelligent biomanufacturing offers a promising pathway to transition PCA from laboratory-scale research to efficient, cost-effective, and scalable green production, setting a model for the industrial biomanufacturing of natural phenolic acids.

## Figures and Tables

**Table 1:** Comparison of strains engineered for PCA production.

Strains	PCA (g/L)	Yield (g/g)	Substrates	Modulating Strategies	Cultivation	References
*E. coli* ATCC 31882	4.25	0.18	Glucose	Overexpression of *DSD* from *P. putida*, and deletion of *aroF*, *aroG*, *tyrR*, *pheA*, *tyrA*, *trpE*	Fed-batch; bioreactor	[77]
*E. coli* MG1655	2.70	0.09	Glucose	Overexpression of *DSD* from *C. glutamicum* and deletion of *aroE*	Batch; test tubes	[67]
*E. coli* BL21 (DE3)	0.64	0.01	Glucose	Co-culture strain A: overexpression of *pobA*. Strain B: overexpression of *aroE*, *aroL*, *aroA*, *aroC*, *ubiC*, *aroG^fbr^*, *aroB*, *aroD*, and *pobR*, deletion of *xylA*, *tyrA*, and *pheA*	Batch; shake flask	[78]
*E. coli* P7	26.70	--	Glucose	Evolution enhanced strain tolerance; expression of *aroE*; reconstruction of the NOG pathway	Fed-batch; bioreactor	[20]
*E. coli* PCA05	46.65	0.23	Glucose	Introduction of ApAroZ^R363A^	Fed-batch; bioreactor	[65]
*E. coli* PCA05	0.49	--	Glucose	Overexpression of *DSD*	Batch; shake flask	[68]
*C. glutamicum*	10.61	--	Methanol and pentose sugars	Deletion of *pcaGH* and *rpe*; overexpression of *xylAB*, *aroG*, *aroB*, *aroD*, and *qsuB*	Batch; shake flask	[71]
*C. glutamicum Pt-Cg7*	63.22	0.35	Glucose	Deletion of *pcaGH*; Overexpression of *tkt*, *aroB*, *aroD*, *qsuB*, *pobA*, and *UbiC*	Fed-batch; bioreactor	[79]
*C. glutamicum* ATCC 21420	1.17	0.008	Glucose	Overexpression of *ubiC* and *pobA*	Fed-batch; bioreactor	[70]
*C. glutamicum* PCA_XYL_ Δ*pyk*	9.60	--	d-xylose	Deletions of *pyk*, overexpression of *xylA_Xc_*, *xylB_Cg_*, *aroB*, *aroD*, *aroF*, *qsuB*, *tkt*	Batch; bioreactor	[72]
*C. glutamicum* LHglc1073	82.70	0.33	Glucose	Overexpression of *aroG^S180F^*, *tkt*, *tal*, *aroA*, *aroD*, *aroE*, *qsuB*, *ubiC*, *pobA*, and *aroCKB*; Deletion of *qsuD*, *pcaGH*, and *poxF*	Fed-batch; bioreactor	[80]
*B. licheniformis* PCA22	21.68	0.54	Glucose	Deletions of *BsdBCD*, overexpression of 3-DHS dehydratase	Fed-batch; bioreactor	[73]
*P. putida* KGVA05	38.80	--	Glucose	overexpression of *quiC*, *aroG^opt^*, *aroQ*, and *aroB*; attenuation of shikimate dehydrogenase	Fed-batch; bioreactor	[81]
*P. putida* PCA1601	2.40	--	lignin hydrolysate	Expression of PobA and VanAB	Fed-batch; bioreactor	[75]
*P. putida* KT2440	21.70	--	Glucose	overexpression of *quiC*, *aroG^opt^*, *tal* Deletion of *pcaGH*, *ppc*, *pyk*, *pykA*, and *aroE*	Fed-batch; bioreactor	[74]

## Data Availability

No new data were created or analyzed in this study. Data sharing is not applicable to this article.

## Abbreviations

PCA, Protocatechuic acid; oxLDL, Oxidized low-density lipoprotein; ALE, adaptive laboratory evolution; DHS, 3-dehydroshikimate; 4-HBA, 4-hydroxybenzoate; EMP, Embden–Meyerhof–Parnas; PPP, pentose phosphate pathways; PEP, phosphoenolpyruvate; E4P, erythrose-4-phosphate; DAHP, 3-deoxy-D-arabino-heptulosonate-7-phosphate; PTS, phosphotransferase system; TCA, tricarboxylic acid.
